# Maternal and neonatal outcomes of pregnancies after metabolic bariatric surgery: a retrospective population-based study

**DOI:** 10.1016/j.lanepe.2025.101263

**Published:** 2025-03-22

**Authors:** Pierre Bel Lassen, Anne-Isabelle Tropeano, Armelle Arnoux, Estelle Lu, Louis Romengas, Sandrine Katsahian, Bérénice Ségrestin, Bénédicte Lelièvre, Delphine Mitanchez, Géraldine Gascoin, Tigran Poghosyan, Andrea Lazzati, Barbara Heude, Jacky Nizard, Sébastien Czernichow, Cécile Ciangura, Claire Rives-Lange

**Affiliations:** a Université Paris Cité and Université Sorbonne Paris Nord, Inserm, INRAE, Center for Research in Epidemiology and StatisticS (CRESS), Paris F-75004, France; b Sorbonne University, Assistance Publique Hôpitaux de Paris, Service de Nutrition, Hôpital Pitié-Salpêtrière, Paris F-75013, France; c Sorbonne Université, Inserm, Nutrition and Obesities: Systemic Approaches, NutriOmics, Research Unit, Paris, France; d Assistance Publique Hôpitaux de Paris, Unité de Recherche Clinique et Service de Santé Numérique, Université Paris Centre, Hôpital Européen Georges Pompidou, Paris F-75015, France; e Assistance Publique Hôpitaux de Paris, Service de Nutrition, Hôpital Européen Georges Pompidou, Paris F-75015, France; f INSERM CIC1418, Epidémiologie Clinique, Hôpital Européen Georges Pompidou, Paris F-75015, France; g INSERM-INRIA-Université paris Cité, UMR 1346, HeKA Team, PariSanté Campus, Paris F-75015, France; h Centre de Recherche en Nutrition Humaine Rhône-Alpes, Univ-Lyon, CarMeN Laboratory, INSERM, INRA, INSA Lyon, Université Claude Bernard Lyon 1, Hospices Civils de Lyon, F-CRIN/FORCE Network, Pierre-Bénite, France; i CHU Angers, Laboratoire de Pharmacologie-toxicologie, Angers F-49933, France; j Department of Neonatology, Bretonneau Hospital, François Rabelais University, Tours F-37000, France; k INSERM UMR_S 938 Centre de Recherche Saint Antoine, Paris F-75012, France; l Department of Neonatal Medicine, Toulouse University Hospital Center, Toulouse, France; m Centre for Epidemiology and Population Health Research, Team SPHERE, Toulouse III University, Toulouse, France; n Assistance Publique Hôpitaux de Paris, Service de Chirurgie Digestive, œsogastrique et Bariatrique, Hôpital Bichat, Université Paris Cité, Inserm UMRS 1149, Paris 75018, France; o Assistance Publique Hôpitaux de Paris, Service de Chirurgie Digestive, Hôpital Avicenne, Sorbonne Paris Nord, Bobigny F-93000, France; p Assistance Publique-Hôpitaux de Paris (AP-HP), Service de Gynécologie Obstétrique, Hôpital Pitié-Salpêtrière, Paris, France; q Inserm U1150, CNRS UMR 7222, Sorbonne Université, Paris, France

**Keywords:** Obesity, Bariatric surgery, Pregnancy, Small-for-gestational age, Prematurity, Neonatal death, Nutrition, Malnutrition, Gastric bypass, Sleeve gastrectomy, Gestational diabetes, Gestational hypertension, Preeclampsia

## Abstract

**Background:**

The incidence of post metabolic bariatric surgery (BS) pregnancies is rising. Previous studies provided conflicting results regarding the risk of prematurity, stillbirth, perinatal death and the optimal time from BS to conception. This study examined maternal and neonatal outcomes of post-BS pregnancies and influencing factors.

**Methods:**

Nationwide retrospective study of all post-BS pregnancies in France from January 1st 2013 to December 31st 2022. We compared 55,941 post-BS pregnancies with 223,712 controls matched on delivery date, parity, age, obesity, hypertension, diabetes, and socio-economic status (1:4 ratio) using generalized estimating equations. We also compared 11,777 post-BS pregnancies with 11,777 pre-BS pregnancies in the same women, using conditional logistic regression. Maternal outcomes included gestational hypertension, preeclampsia, and gestational diabetes. Neonatal outcomes included small-for-gestational-age (SGA), prematurity, stillbirth, and perinatal death. We tested for interactions with BS type, BS to pregnancy time interval and malnutrition.

**Findings:**

Post-BS pregnancies were associated with reduced risk of gestational hypertension (odds ratio [OR] 0.57 [95% CI 0.53–0.62]), preeclampsia (OR 0.59 [0.55–0.64]), and gestational diabetes (OR 0.64 [0.62–0.66]) as compared with control. Similar but stronger risk reductions were observed compared with pre-BS pregnancies. Risk of SGA was increased (OR 1.74 [1.68–1.79]) as compared with controls and pre-BS pregnancies (OR 1.88 [1.64–2.16]). Risk of prematurity was increased (OR 1.27 [1.22–1.31]) as compared with controls but not pre-BS pregnancies (OR 0.95 [0.85–1.06]). Compared with controls, risk of stillbirth was increased (OR 1.2 [1.06–1.35]), mediated by SGA, as was perinatal death (OR 1.5 [1.13–1.99]), mediated by both prematurity and SGA. Increased SGA risk compared to controls was higher with malnutrition (OR: 2.38 [1.96, 2.88], p_interaction_ <0.0001), with <6 months (OR: 1.95 [1.72, 2.21], p_interaction_ = 0.01) or 6–12 months between BS and pregnancy (OR: 1.86 [1.70, 2.04], p_interaction_ = 0.02) and with gastric bypass (OR: 1.88 [1.77–2.00], p_interaction_ = 0.027). Increased prematurity risk compared to controls was higher with malnutrition (2.45 [1.99, 3.00], p_interaction_ <0.0001) and gastric bypass (OR: 1.46 [1.36–1.57], p_interaction_ = 0.0003).

**Interpretation:**

Post-BS compared with pre-BS or control pregnancies were associated with reduced risk of maternal adverse outcomes but increased risk of neonatal adverse events. The risks of SGA and prematurity are higher with shorter intervals between BS and conception, gastric bypass, and malnutrition. Post-BS pregnancies could be considered high risk, requiring close nutritional and obstetrical monitoring.

**Funding:**

Support from INSERM and the French Ministry of Health (Messidore 2022 n°97).

**Research in context**

**Evidence before this study**

Bariatric surgery (BS) is associated with reduced risks of gestational diabetes, hypertensive disorders, and large-for-gestational-age neonates in post-BS pregnancies. BS increases the risk of small-for-gestational-age (SGA) neonates, with conflicting data on the risk of prematurity, stillbirth, and perinatal death. The impact of different BS procedures, the optimal time to conceive post-surgery, and their effects on maternal and neonatal outcomes require further large-scale investigation. Literature search for English language publications was performed using PubMed and Google Scholar covering the period from 01/01/2000 to 01/09/2024 using the terms *bariatric surgery*, *pregnancy*, and *obesity*.

**Added value of this study**

This large population-based study confirms lower risks of gestational hypertension, preeclampsia, and gestational diabetes, and higher risk of SGA in post-BS pregnancies. Prematurity risk is increased in post-BS pregnancies compared to controls but not compared to pre-BS pregnancies in the same woman. Stillbirth and perinatal death risks are higher in post-BS pregnancies compared to controls, mediated respectively by SGA and SGA and prematurity. The risks of SGA and prematurity are higher with shorter intervals between BS and conception, gastric bypass, and malnutrition.

**Implications of all the available evidence**

Post-BS pregnancies could be considered high-risk, necessitating close nutritional and obstetric monitoring to mitigate potential neonatal complications. The increased neonatal risks and the greater benefits for LGA and maternal morbidity with gastric bypass, are to consider in the risk-benefit balance assessment of the BS type in women of childbearing age.

## Introduction

Severe obesity prevalence is rising^1^ and it significantly affects reproductive health, increasing the risk of adverse pregnancy outcomes such as early pregnancy loss, gestational diabetes, excessive fetal growth, hypertensive disorders, cesarean deliveries, preterm births, and stillbirths.^2^^,^^3^ Metabolic bariatric surgery (BS) is the most effective and permanent method for substantial weight loss in people living with severe obesity and improvement of obesity-related comorbidities^4^ but can be associated with nutritional complications.^5^^,^^6^ Up to 720,000 such interventions are performed annually worldwide, about 80% of these procedures in women, most of childbearing age.^7^ Therefore, post-BS pregnancies are becoming increasingly common.^8^ BS can reduce the risk of gestational diabetes,^9^, ^10^, ^11^ large-for-gestational-age (LGA) neonates, and hypertensive disorders^9^, ^10^, ^11^, ^12^ and improve delivery outcomes.^13^ However, BS can lead to malnutrition and micronutrient deficiencies, with some evidence of unfavorable pregnancy outcomes. Consistent reports have shown increased risk of small-for-gestational-age (SGA; i.e., birth weight <10th percentile) neonates^9^, ^10^, ^11^^,^^14^, ^15^, ^16^ after BS. The risk of prematurity in post-BS pregnancies is still debated because of conflicting studies.^9^, ^10^, ^11^^,^^15^, ^16^, ^17^, ^18^ A previous study of stillbirth and perinatal death showed trends of increased risk in post-BS pregnancies.^9^

Different BS procedures, such as gastric bypass and sleeve gastrectomy, may have distinct impacts on maternal nutrition, fetal growth, overall adverse pregnancy and neonatal outcomes. In most studies, the more malabsorptive procedures such as biliopancreatic diversion seem associated with low birth weight,^14^^,^^17^^,^^19^ although larger samples are needed to clearly distinguish the effects of the different surgery types on the risk of SGA neonates and other adverse pregnancy and neonatal outcomes. Similarly, the optimal time from BS to conception to minimize adverse pregnancy outcomes is still debated. Institutions and expert consortia recommend delaying pregnancy for at least 12–18 months after surgery,^20^, ^21^, ^22^, ^23^ but a recent systematic review suggested that early pregnancy after BS (≤12 months) was not related to adverse neonatal outcomes.^24^ However, most studies had a small sample size (n < 100) and were probably underpowered to detect any association.

Previous results have also varied in the type of the control population used,^17^ whether matched with non-BS control pregnancies or pre-BS pregnancies in the same woman. The former is useful to assess the risk of a post-BS pregnancy compared to women from the general population with matched BMI, whereas the latter evaluates the risk–benefit balance of having a pregnancy after vs. before BS and is a good model to study whether maternal and fetal outcomes are attributable to BS. To date, these analyses have not been combined in the same study.

We conducted a large population-based study to evaluate the risks and benefits of post-BS pregnancies on adverse pregnancy outcomes (≥22 weeks of gestation [WG]), maternal and neonatal outcomes. A secondary objective was to assess the association of surgery type, timing and nutritional factors with these outcomes.

## Methods

This study is part of the NUMASURG project investigating the role of maternal nutritional status during pregnancy on health outcomes using nutritional biomarkers.^25^ Specifically, this study examined the associations between a history of BS and pregnancy and child outcomes using data from a large-scale medical database.

### Data source

Data were extracted from the French National Health Data System (SNDS), covering 99% of the population in France (>66 million people). This database has linked newborns to their mothers since 2012 and includes demographic data, International Classification of Diseases, 10th revision (ICD-10) diagnoses, hospital procedures, and healthcare consumption covered by the national health insurance in France. The project has been registered with the SNDS access authority and the Health Data Hub (no. 16515049). Ethical approval and individual informed consent was not required, as the study exclusively used SNDS data,. Compliance with data protection and patient rights is ensured by the Health Data Hub and the National Health Insurance Fund, which oversee the processing and governance of SNDS data. Procedures.

The database, dating back to 2005, was used to identify prior BSs and pregnancies. Sleeve gastrectomy, gastric bypass, adjustable gastric band and bilio-pancreatic derivation were identified by using procedure codes from the French National Procedures Classification linked to obesity diagnoses (ICD-10 code E66).^10^^,^^26^ A previously developed algorithm identified pregnancies ≥22 WG.^27^ The pregnancy outcome date was defined as the date of delivery or termination or, if missing, the date of admission for pregnancy completion. The conception date was calculated by using the outcome date and gestational age or, if missing, the last menstrual period date recorded by the physician.

### Study design and participants

**Fig. 1:**
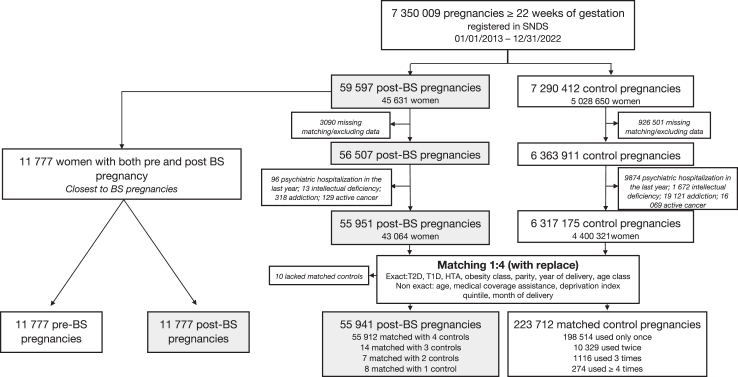
**Study flow chart.** BS: metabolic bariatric surgery; T2D: type 2 diabetes mellitus; T1D: type 1 diabetes; HTA: arterial hypertension; SNDS: French National Health Data System.

We included all pregnancies ≥22 WG from January 1, 2013, to December 31, 2022 in women with prior BS (Fig. 1). These post-BS pregnancies were matched with control pregnancies, with up to 4 controls per case with replacement (0.1% of cases were matched with less than 4 when exact matching was not possible). Matching involved using the nearest-neighbor method on a propensity score with the R *matchit* package. Exact matching variables were age categories (<25, 25–29, 30–34, ≥35 years), parity (nulliparous or parous), pregnancy BMI class (<30, 30–39, 40–49, ≥50 kg/m^2^), type 1 and type 2 diabetes, hypertension, and delivery year. Non-exact matching variables included age (continuous), health coverage assistance, deprivation index quintiles, and delivery month (Supplemental appendix). To avoid selection bias, we excluded from both groups women with contraindications for BS, such as recent psychiatric hospitalization, intellectual disability, addiction, and active cancer. Additionally, we excluded women with missing data for any matching criterion (Fig. 1).

Among post-BS pregnancies, we identified women who had also at least one pregnancy before BS and no prior BS for these pre-BS pregnancies. We selected the closest pregnancies before and after BS for analysis, with each pre-BS pregnancy serving as a control for the post-BS pregnancy. We performed a counterfactual analysis comparing 2 distinct pre-BS pregnancies in the same woman, which is detailed in the Supplemental appendix.

Methods for defining covariates and matching variables are developed in further detail in the Supplemental appendix. Briefly, comorbidities of mothers before pregnancy (type 1 and 2 diabetes, hypertension, sleep apnea), socioeconomic status (social deprivation index and health coverage assistance), exclusion criteria, and matching variables were exhaustively identified in the SNDS for the selected population. However, information on obesity status relies on ICD-10 coding at pregnancy and may be missing in some cases. We used a machine learning approach (random forest algorithms) trained on exhaustive ICD-10 obesity coding centers to impute missing data for this variable (Supplementary appendix). These algorithms were applied to post-BS pregnancy data before matching, reestablishing a higher obesity proportion in this population and therefore in matched controls. Malnutrition status was defined if artificial nutrition was prescribed or in case of hospitalization for malnutrition or severe eating disorder from BS date to delivery (detailed in Supplementary appendix).

### Outcomes

Mother-to-child linkage and algorithms for identifying each outcome are detailed in the Supplementary appendix. Adverse pregnancy maternal outcomes included gestational diabetes, gestational hypertension, preeclampsia or eclampsia and pregnancy termination. Neonatal outcomes were obtained by linking each mother to her child in the database.^27^^,^^28^ SGA was defined as birth weight <10th percentile (moderate: 3–9th; severe: <3rd). LGA was defined as birth weight ≥90th percentile (moderate: 90–96th; severe: ≥97th). Birth weight percentiles adjusted for gestational age and sex were determined by using Audipog reference curves.^10^ Prematurity was defined as a live birth <37 WG (moderate: 32–36 + 6, severe: 28–31 + 6, extreme: <28). Stillbirth was defined as the birth of a deceased neonate, and perinatal mortality included all live births followed by the death of a child within the first 7 days of life.

### Statistical analysis

The statistical unit was a pregnancy, allowing multiple contributions per woman. For the post-BS vs. matched controls analysis, we used generalized estimating equations clustering by mother's ID and assuming an exchangeable correlation structure with the R *geepack* package to estimate risks of adverse outcomes. Models were additionally adjusted for age (continuous), health coverage assistance, deprivation index quintiles, pregnancy obesity status, sleep apnea syndrome, and medically assisted reproduction. Weights were applied: post-BS pregnancies had a weight of 1, and control pregnancies were weighted inversely to their frequency.

Mediation analyses for stillbirth and perinatal death, focusing on SGA and prematurity, involved using the R *mediation* package.

Interactions for BS type, time from BS to pregnancy, pre-BS BMI, and malnutrition status were assessed, and subgroup analyses stratified by these variables were performed in the post-BS vs. control population. To account for not only the OR but also the proportion differences they represent, we have specified in the Supplementary Tables, in addition to the ORs, the percentage differences.

For the post-BS vs. pre-BS and counterfactual analyses, conditional logistic regression models involved using the R *clogit* package.

Finally, we performed a lasso regularization analysis in all post-BS pregnancies to identify the main predictors of SGA and prematurity in this population and the importance of each predictor by using the R *elasticnet* package.

Analyses involved using RStudio Pro 2022.12.0. p < 0.05 was considered statistically significant. Multiple comparisons were adjusted with the false discovery rate method, and confidence intervals (CIs) were enlarged in proportion to the p-value increase after adjustment. Results are presented as odds ratios (OR) with 95% CIs. To account for not only the OR but also the proportion differences they represent, we have specified in the Supplementary Tables, in addition to the ORs, the percentage differences.

### Role of the funding source

The funder of the study had no role in study design, data collection, data analysis, data interpretation, or writing of the report.

## Results

The flow chart of population selection and matching is in Fig. 1.

### Post-BS vs. matched control pregnancies

From all pregnancies reaching at least 22 WG during the study period registered in the database, 55,941/7,350,009 (0.8%) occurred in women with a history of BS (Table 1). In 2022, post-BS pregnancies represented 1.6% of all pregnancies. They were matched with 223,712 controls (Fig. 1). The mean (SD) age of women with post-BS pregnancies was 31.2 (4.9) years, and the prevalence of obesity at pregnancy (after imputation) was 26.8%, including a prevalence of 7.5% for grade 3 obesity (BMI ≥ 40 kg/m^2^). The most frequent BS interventions were sleeve gastrectomy (68.3%) and gastric bypass (23.6%), with a mean time from BS to pregnancy of 2.9 (1.9) years. Medically assisted reproduction and treated sleep apnea syndrome were more common in post-BS than control pregnancies and were therefore adjusted for in further analysis. After matching between post-BS and control pregnancies, the 2 groups did not significantly differ in age, health coverage assistance, deprivation index quintile, obesity at pregnancy, diabetes, hypertension, or parity and the post-matching standardized difference is low (Supplementary Fig. S1).

**Table 1:** Maternal characteristics of post-BS pregnancies (vs. control and vs. pre-BS pregnancies).

Characteristics	Post-BS vs. matched control pregnancies			Post-BS vs. pre-BS pregnancies (in the same mother)		
	Post-BS	Control	p value	Post-BS	Pre-BS	p value
	N = 55,941	N = 223,712		N = 11,777	N = 11,777	
Type of surgery, n (%)						
Sleeve gastrectomy	38,198 (68.3)	–	–	8727 (74.1)	–	–
Gastric bypass	13,193 (23.6)	–	–	2323 (19.2)	–	–
Adjustable gastric band	4456 (8.0)	–	–	704 (6.0)	–	–
Bilio-pancreatic derivation	94 (0.2)	–	–	23 (0.2)	–	–
BMI class before BS, n (%)						
30–39 kg/m^2^	17,699 (31.6)					
40–49 kg/m^2^	33,252 (59.4)					
≥50 kg/m^2^	4983 (8.9)					
Surgery to pregnancy interval, n (%)						
<6 months	2905 (5.2)	–	–	1026 (8.7)	–	–
6–12 months	5870 (10.5)	–	–	1901 (16.1)	–	–
1–2 years	13,498 (24.1)	–	–	4044 (34.3)	–	–
2–5 years	24,783 (44.3)	–	–	4333 (36.8)	–	–
≥5 years	8885 (15.9)	–	–	473 (4.0)	–	–
Age, mean (SD), y	31.2 (4.9)	31.2 (4.9)	0.38	31.4 (4.6)	26.5 (4.4)	<0.0001
Age categories, n (%)			1.00			<0.0001
<25 y	4619 (8.3)	18,473 (8.3)	1.00	692 (5.9)	4072 (34.6)	<0.0001
25–30 y	16,971 (30.3)	67,873 (30.3)	1.00	3546 (30.1)	4835 (41.1)	<0.0001
30–35 y	19,802 (35.4)	79,183 (35.4)	1.00	4605 (39.1)	2374 (20.2)	<0.0001
>35 y	14,549 (26.0)	58,183 (26.0)	1.00	2934 (24.9)	496 (4.2)	<0.0001
Health coverage assistance, n (%)	15,764 (28.2)	62,258 (27.8)	0.10	4214 (36.3)	4148 (36.5)	0.74
Deprivation index quintiles, n (%)			0.61			0.18
1	7156 (12.8)	28,246 (12.6)	0.29	1378 (12.1)	1231 (11.1)	0.01
2	10,416 (18.6)	41,724 (18.7)	0.87	1981 (17.5)	1921 (17.4)	0.87
3	11,321 (20.2)	45,020 (20.1)	0.55	2326 (20.5)	2272 (20.5)	0.87
4	12,347 (22.1)	49,290 (22.0)	0.85	2511 (22.1)	2481 (22.4)	0.97
5	14,701 (26.3)	59,432 (26.6)	0.17	3152 (27.8)	3156 (28.5)	0.72
Obesity at pregnancy, n (%)	14,996 (26.8)	59,932 (26.8)	0.94	3298 (28.0)	11,765 (99.9)	<0.0001
30–39 kg/m2	10,789 (19.2)	43,118 (19.2)	0.93	713 (6.1)	3909 (33.2)	<0.0001
≥40 kg/m2	4207 (7.5)	16,814 (7.5)	0.98	4207 (7.5)	7856 (66.7)	<0.0001
Diabetes (treated) at pregnancy, n (%)						
Type 1	95 (0.2)	349 (0.2)	0.50	19 (0.2)	17 (0.1)	0.87
Type 2	484 (0.9)	1915 (0.9)	0.85	84 (0.7)	138 (1.2)	0.0005
Hypertension (treated) at pregnancy, n (%)	1378 (2.5)	5469 (2.4)	0.81	250 (2.1)	197 (1.7)	0.01
Sleep apnea syndrome (treated), n (%)	1900 (3.4)	698 (0.3)	<0.0001	600 (5.1)	58 (0.5)	<0.0001
Nulliparous, n (%)	16,825 (30.1)	67,280 (30.1)	0.99	0 (0)	6152 (52.2)	
Medically assisted reproduction, n (%)	4460 (8.0)	15,196 (6.8)	<0.0001	525 (4.5)	787 (6.7)	<0.0001

For post-BS vs. matched controls, continuous variables were compared by Student *t* test and categorical variables by chi-squared test; For post-BS vs. pre-BS, continuous variables were compared by paired t test and categorical variables Mc Nemar chi-squared test. BS: Metabolic bariatric surgery. Ethnicity data is not available in the SNDS database.

### Post-BS vs. pre-BS pregnancies

Among women with post-BS pregnancies, 11,777 had both one pregnancy before and one after BS. The mean (SD) time from pre-BS to post BS pregnancy was 4.8 (1.7) years (Table 1).

### Adverse pregnancy and neonatal outcomes

#### Gestational hypertension, preeclampsia and gestational diabetes

**Fig. 2:**
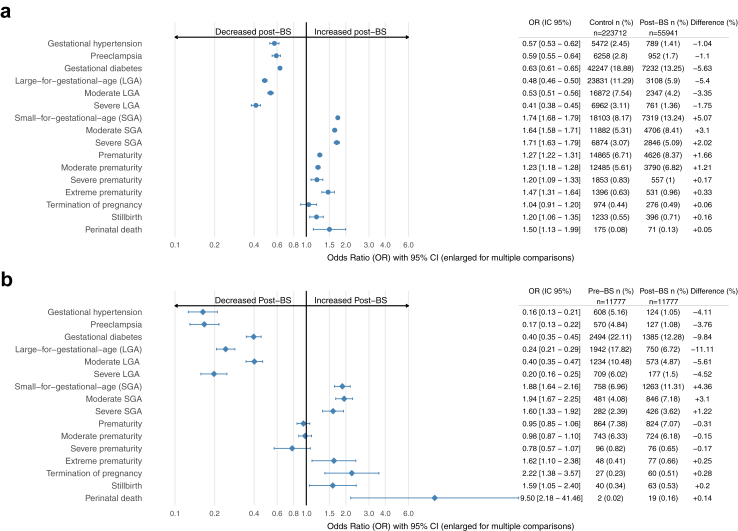
**Adverse pregnancy and neonatal outcomes associated with post-bariatric surgery (BS) pregnancies.** Occurrence of adverse pregnancy and neonatal outcomes of post-BS vs. matched control pregnancies **(a)** and pre-BS control pregnancies **(b)**. Odds ratios are calculated from generalized estimated equations adjusted for age (continuous), health coverage assistance, deprivation index quintiles, pregnancy obesity status, sleep apnea syndrome and medically assisted reproduction for that pregnancy in **a** and from conditional logistic regression with the mother ID as a stratum in **b**. Confidence intervals are enlarged proportionally to the p-value increase after multiple comparison adjustment by using the false discovery rate method. BS: metabolic bariatric surgery; W: weeks of gestation.

As compared with matched control pregnancies, post-BS pregnancies were associated with decreased risk of gestational hypertension (1.4% vs. 2.4%, OR: 0.57 [0.53–0.62]), preeclampsia (1.7% vs. 2.8%, OR: 0.59 [0.55–0.64]), and gestational diabetes (13.2% vs. 18.9%, OR: 0.64 [0.62–0.66]) (Fig. 2). The risk reductions were greater when comparing post-BS to pre-BS pregnancies in the same mother: gestational hypertension (1.1% vs. 5.2%, OR: 0.16 [0.12–0.21]), preeclampsia (1.1% vs. 4.8%, OR: 0.17 [0.13–0.22]), and gestational diabetes (12.3% vs. 22.1%, OR: 0.40 [0.32–0.42]).

#### Birth weight

Mean (SD) birth weight was lower in post-BS than matched control pregnancies (3127 [564] g vs. 3309 [549] g; p < 0.0001). As compared with matched control pregnancies, post-BS pregnancies were associated with increased risk of SGA neonates (13.3% vs. 8.2%, OR: 1.74 [1.68–1.79]), affecting significantly both moderate (+3.1%) and severe (+2.0%) SGA. We found similar results when comparing post-BS to pre-BS pregnancies in the same mother. However, the risk of LGA neonates was decreased in post-BS vs. control pregnancies (5.9% vs. 11.3%, OR: 0.48 [0.46–0.50]) and pre-BS pregnancies in the same mother (OR: 0.24 [0.21–0.29]).

#### Prematurity

As compared with matched control pregnancies, post-BS pregnancies were associated with increased risk of overall prematurity (8.4% vs. 6.7%, OR: 1.27 [1.22–1.31]), with significant increases in moderate (+1.2%), severe (+0.2%), and extreme (+0.3%) prematurity. Usual causes of prematurity (preeclampsia, chorioamniotitis, abruptio, placenta praevia or oligo-amnios) did not account for this increase (i.e., were not significantly higher in post-BS than control pregnancies; data not shown). The overall incidence of prematurity did not differ between pre- and post-BS pregnancies in the same woman.

#### Stillbirth and perinatal death

As compared with matched control pregnancies, post-BS pregnancies were associated with increased risk of stillbirth (0.71% vs. 0.55%, OR: 1.2 [1.06–1.35], p = 0.003) and perinatal death (0.13% vs. 0.08%, OR: 1.5 [1.13–2.00], p = 0.004). Mediation analysis showed that BS had no significant direct effect on stillbirth. However, BS was linked to SGA, which in turn was associated with stillbirth. The mediation effect accounted for 38.6% and was significant (p < 0.0001). Similarly, BS had no direct effect on perinatal death, but it was associated with prematurity and SGA, both of which were linked to perinatal death. The mediation effects were 61.1% for prematurity (p < 0.0001) and 31.1% for SGA (p = 0.02).

Risk of stillbirth was significantly increased in post-vs. pre-BS pregnancies in the same mother (OR: 1.59 [1.05–2.4]); the small number of events of perinatal death (n = 2) in pre-BS pregnancies did not allow for conclusions for this outcome.

#### Termination of pregnancy

We found no significant association between post-BS pregnancy and risk of termination of pregnancy as compared with matched controls. However, when comparing post- and pre-BS pregnancies in the same mother, the risk was increased (0.5% vs. 0.2%; OR 2.22 [1.38–3.58]).

### Subgroup and interaction analyses

These analyses were performed solely in the post-BS vs. matched control population.

#### BS type

**Fig. 3:**
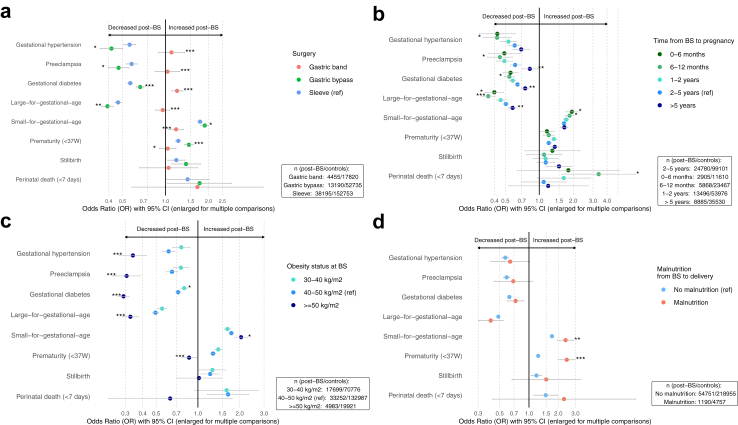
**Surgical and nutritional factors associated with adverse pregnancy and neonatal outcomes.** Occurrence of adverse pregnancy and neonatal outcomes of post-BS vs. matched control pregnancies (significant in the whole population) stratified by BS type **(a),** time from BS to pregnancy **(b),** obesity class before BS **(c)** and malnutrition status from BS to delivery **(d)**. Odds ratios are calculated from generalized estimated equations adjusted for age (continuous), health coverage assistance, deprivation index quintiles, pregnancy obesity status, sleep apnea syndrome and medically assisted reproduction for that pregnancy. Confidence intervals are enlarged proportionally to the p-value increase after multiple comparison adjustment by using the false discovery rate method. Interactions for each subgroup were tested with sleeve gastrectomy as a reference in **a**, 2–5 years post-BS in **b**, BMI 30–40 kg/m^2^ in **c** and no malnutrition from BS to pregnancy in **d**. ∗ p_interaction_ <0.05; ∗∗ p_interaction_ <0.01; ∗∗∗ p_interaction_ <0.001. False discovery rate multiple comparison adjustment was applied for calculating p_interaction_ values. BS: metabolic bariatric surgery; W: weeks after last menstrual period; ref: reference.

We found significant interactions for type of BS concerning risk of gestational hypertension, preeclampsia, gestational diabetes and LGA (p_interaction_ <0.0001) (Fig. 3A, Supplementary Table S1). Protective associations for these conditions were sleeve gastrectomy and gastric bypass but not adjustable gastric band. Protection was greater for gastric bypass than sleeve gastrectomy for gestational hypertension, preeclampsia, and LGA, whereas the OR for sleeve gastrectomy was lower for gestational diabetes. The increased risk of SGA for post-BS vs. matched control pregnancies was significant for all surgery types, with the highest risk for gastric bypass (OR: 1.88 [1.77–2.00]) and the lowest for adjustable gastric band (OR: 1.19 [1.02–1.38]) as compared with sleeve gastrectomy (OR: 1.74 [1.68–1.81]; respectively p_interaction_ = 0.027 and p_interaction_<0.0001). The risk of prematurity was increased with gastric bypass and sleeve gastrectomy but not adjustable gastric band, with a greater risk for gastric bypass than sleeve gastrectomy (OR: 1.46 [1.36–1.57] vs. 1.23 [1.17–1.28]; p_interaction_ <0.0001).

#### Time from BS to pregnancy

Interaction tests revealed significant differences in risk of SGA according to time from BS to pregnancy (Fig. 3B, Supplementary Table S2). The risk of SGA was increased if pregnancy occurred within 6 months post-BS (OR: 1.95 [1.72, 2.21]) and within 6–12 months (OR: 1.86 [1.70, 2.04]) as compared with 2–5 years (OR: 1.66 [1.58, 1.73]) (respectively p_interaction_ = 0.01 and p_interaction_ = 0.02). Similarly, protective associations were higher for LGA during these intervals. The protective effects for preeclampsia, gestational diabetes, and LGA were lower if pregnancy occurred >5 years after BS.

#### Pre-BS BMI class

The interaction test indicated significant differences across pre-BS BMI classes concerning the outcomes studied (p_interaction_ <0.0001) (Fig. 3C, Supplementary Table S3). The subgroup of pregnancies in women with pre-BS BMI >50 kg/m^2^ exhibited more protective associations for gestational hypertension, gestational diabetes, preeclampsia, LGA, and prematurity but also more increased risk of SGA.

#### Malnutrition from BS to delivery

Interaction tests indicated significant associations between malnutrition from BS to delivery and risk of SGA and prematurity (p_interaction_ <0.001) (Fig. 3D, Supplementary Table S4). Malnutrition occurred in 1190 cases (2.1%) of post-BS pregnancies. The risks of SGA and prematurity were significantly higher in this subgroup than in those without malnutrition (OR for SGA: 2.38 [1.96, 2.88] vs. 1.72 [1.67, 1.78]; OR for prematurity: 2.45 [1.99, 3.00] vs. 1.24 [1.20, 1.29]).

### Most important factors associated with SGA and prematurity

**Fig. 4:**
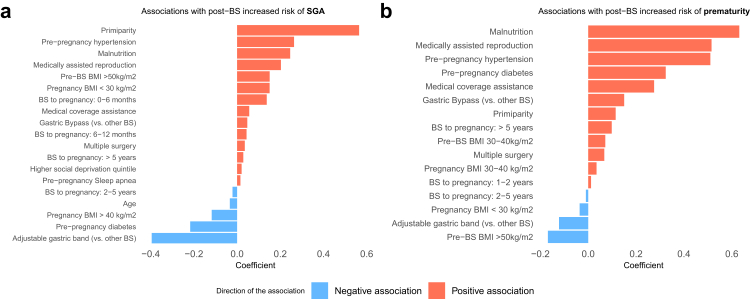
**Main factors associated with small-for-gestational-age (SGA) and prematurity in post-BS pregnancies.** Factors selected in a lasso regularization model associated with SGA **(a)** and prematurity **(b),** among post-BS pregnancies. Beta coefficients from the regularization model are displayed. A negative coefficient implies a protective association, and a positive coefficient implies a risk association. BS: metabolic bariatric surgery.

Lasso regularization models indicated the main predictors associated with SGA (Fig. 4A) and prematurity (Fig. 4B) among post-BS pregnancies (Fig. 4). The 5 main factors associated with SGA were primiparity, pre-pregnancy hypertension and malnutrition (risk associations), gastric banding and pre-pregnancy diabetes (protective associations). The 5 main factors associated with prematurity were malnutrition, medically assisted reproduction, pre-pregnancy hypertension, pre-pregnancy diabetes and medical coverage assistance (risk associations). Multiple surgeries were among the factors associated with increased risk of SGA and prematurity.

## Discussion

In this large real-life nationwide study, post-BS pregnancies compared with matched control pregnancies from the general population and with pre-BS pregnancies from the same mother showed reduced risk of gestational hypertension, preeclampsia, gestational diabetes and LGA but increased risk of SGA. The risk of prematurity was higher with post-BS than control pregnancies but not when comparing infants born from the same woman pre- and post-BS. Additionally, post-BS pregnancies were associated with increased risk of stillbirth and perinatal death. However, these associations were mediated by SGA and by prematurity and SGA, respectively.

Two separate analyses were conducted: an external comparison between post-BS pregnancies and matched controls, and an internal comparison between post-BS pregnancies and pre-BS pregnancies in the same woman. The internal model avoids bias caused by inter-individual variation not accounted for by matching. However, in pre-BS pregnancies, women are younger, have a higher BMI, and nearly half are nulliparous, unlike in post-BS pregnancies (by design). On the other hand, the external model accounts for age, parity, and BMI.

The protective effects for gestational hypertension, preeclampsia, gestational diabetes, and LGA are consistent with previous reports.^9^, ^10^, ^11^ Associations were more pronounced when comparing post-BS and pre-BS pregnancies in the same woman. This can be explained by the higher BMI in pre-BS pregnancies which are therefore at higher risk of metabolic complications than matched controls. In this population, some of the risk reductions could be attributed to the higher frequency of primiparity in pre-vs. post-BS pregnancies, a known risk factor for gestational hypertension and preeclampsia.^29^^,^^30^ However, the ORs found in the counterfactual analysis, which reflect the contribution of older maternal age and greater parity, do not fully account for the protective associations. Obesity is a major risk factor for all of these adverse pregnancy outcomes,^29^^,^^31^ and BS-induced weight loss likely explains the risk reduction.

Our study confirms the increased risk of SGA in post-BS women, in line with previous findings.^9^, ^10^, ^11^^,^^32^, ^33^, ^34^ The risk of adverse perinatal outcomes in neonates is twice as high with severe SGA (<3rd percentile).^35^ Importantly, our sample size allowed for separating moderate and severe SGA, and we observed similar increased risks for both conditions.

Our findings clarify the associations between post-BS pregnancies and pre-term birth, which was found in some^15^^,^^17^^,^^36^ but not all studies.^9^^,^^11^ We found a higher risk of overall prematurity in post-BS than matched control pregnancies but not when comparing post-BS and pre-BS pregnancies in the same woman. The post-BS vs. pre-BS model is not confounded by bias due to insufficient quality of matching with control pregnancies. On the other hand it is possible that the BS-related increased risk of prematurity in this population is counterbalanced by increased risk of prematurity in nulliparous women^37^ which are by design absent in the post-BS population.

We also observed an association between post-BS pregnancies and increased risk of stillbirth and perinatal death. These findings, not previously reported in a single study, were detectable because of our large sample size. Indeed, a study with a similar design to ours but with 100 times fewer cases reported similar positive ORs for stillbirth and neonatal death that did not reach statistical significance.^9^ Increased perinatal mortality was also reported in a meta-analyses of observational studies.^15^ Although our results were statistically significant, the risk difference was small, representing a limited number of cases. Importantly, BS, per se, was not directly linked with increased neonatal mortality, but the association was mediated by SGA and prematurity. This observation highlights the importance of close monitoring and prevention of insufficient weight gain in the case of post-BS pregnancies because they can lead to rare but serious consequences.

We could evaluate the impact of surgery type, time from BS to pregnancy, pre-BS BMI class and malnutrition on the associations between post-BS pregnancies and adverse pregnancy and neonatal outcomes. The risk reductions for gestational hypertension, preeclampsia and LGA were greater with gastric bypass vs. sleeve gastrectomy. Conversely, risk of SGA and prematurity was increased more with gastric bypass than sleeve gastrectomy. This finding could be explained by the greater weight loss observed after gastric bypass^38^ or by the maternal nutritional status, which may be more affected by the intestinal bypass induced by this procedure. Adjustable gastric band is also associated with increased risk of SGA but less so than sleeve gastrectomy and with no benefits on reductions of gestational hypertension, preeclampsia and LGA.

The optimal time from BS to pregnancy remains debated. Most guidelines recommend waiting 12–18 months after BS before conceiving,^20^^,^^21^^,^^23^ but this was challenged by a recent systematic review.^24^ Our study clarifies this point by showing that the risk of SGA was higher within 0–12 months than 2–5 years post-BS. We found no difference for the 1- to 2-year period. In addition, the reduction in risk of gestational hypertension, diabetes, and preeclampsia was less when pregnancy occurred >5 years post-BS, likely due to maternal weight regain.

Pre-BS BMI >50 kg/m^2^ was associated with protection against gestational hypertension, gestational diabetes, preeclampsia and prematurity but increased risk of SGA. Because matching was performed on pregnancy BMI, this greater protection could be explained by the high post-BS weight loss in women with increased pre-BS BMI, which prevents the complications associated with severe obesity but increases the risk of SGA.

Malnutrition, although reported in a small proportion of post-BS pregnancies, was associated with double the risk of both SGA and prematurity. This observation suggests that women with malnutrition should receive particular attention ideally before but at least during pregnancy and highlights the importance of ensuring adequate nutritional intake in post-BS pregnant women.

Several pathogenic mechanisms may underlie the increased risk of SGA after BS. Our results suggest that intestinal bypass and malnutrition status in the mother are key aspects. Maternal hypoglycemia was also suggested as a potential mechanism, but we were not able to assess this in the present study.^39^

The decrease in the use of medically assisted reproduction in post-BS vs. pre-BS pregnancies seems to indicate that post-BS induced weight loss induced leads to a significant improvement in fertility, sufficient to outweigh the effects of increasing maternal age and the removal of BMI-related barriers to accessing medically assisted reproduction.

Several limitations of our study need to be mentioned. Although the SNDS database provides comprehensive data, factors such as ethnicity, quality of nutritional follow-up, smoking and alcohol consumption are not recorded, which leads to potential residual differences between post-BS and control women despite matching. Also, BMI is not reported as a continuous variable and only BMI categories were available in the database, which limited calculating the post-BS weight loss. In addition, except for pre-BS BMI, BMI is also often partially coded, thereby potentially underestimating post-BS obesity. To address this situation, we used machine learning to impute BMI in post-BS women. Although the specificity of the imputation model was high, a small proportion of post-BS women may have been imputed as with obesity when it was not the case. Nutritional status could not be accurately measured because vitamin deficiencies and anemia particularly are not well reported in the database. Because of this limited data, we could only detect in our study the most severe cases of malnutrition where hospitalization or artificial nutrition was necessary. We were not able to differentiate between Roux-en-Y gastric bypass and one anastomosis gastric bypass in our data but we expect that they are mostly Roux-en-Y since one anastomosis gastric bypass was removed from health care reimbursement in France in 2019. Cases with band removal can’t be eliminated as there is no specific code for band removal but the highest possible rate of band removal was calculated to 17.2%. The subgroup and interaction analyses were solely performed in post-BS vs. matched control population because it was the one with sufficient sample to evaluate both rare events and rather rare interaction variables. Lastly, we restricted to pregnancies beyond 22 WG and therefore we did not assess the risk of miscarriage or other first trimester loss and of malformations. The decrease in the use of medically assisted reproduction in post-BS vs. pre-BS pregnancies seems to indicate that post-BS induced weight loss induced leads to a significant improvement in fertility, sufficient to outweigh the effects of increasing maternal age and the removal of BMI-related barriers to accessing medically assisted reproduction.

The strengths of our study include its unprecedented sample size, 25 times larger than previous works, and its comprehensive population-level approach for inclusions, outcomes, and risk factors of adverse pregnancy outcomes. This extensive scope allowed for investigating rarer outcomes such as stillbirths and perinatal mortality, a clear distinction of BS type and intervals between BS and pregnancy impacts, and is the first report on malnutrition effects. It also allowed for high-quality matching of BS-exposed women with controls. Additionally, including both post-BS vs. control and post-BS vs. pre-BS comparisons offered valuable insights into the risks of post-BS pregnancies and helped assess the risk–benefit balance of pregnancy timing from both healthcare and patient perspectives.

Our findings may help healthcare professionals to inform patients considering pregnancy after BS and serve to improve current recommendations in this field.^20^^,^^21^^,^^23^ Although most pregnancy outcomes are improved at an individual level after BS, guidelines could alert professionals to consider these pregnancies as high-risk, and the need to offer a reinforced monitoring of fetal growth and of maternal nutritional status. As poor outcomes are mainly mediated by SGA and prematurity, and SGA and prematurity strongly associated with maternal malnutrition, research should focus on investigating nutritional mechanisms and optimizing maternal nutritional care.

### Conclusions

We found that post-BS pregnancies protected against gestational hypertension, diabetes, and preeclampsia, but risk of SGA and prematurity was increased, potentially increasing risks of stillbirth and perinatal death. Post-BS pregnancies could be considered as high risk, requiring particular nutritional attention and enhanced obstetrical surveillance. Studies with longer follow-up of neonates are needed to assess long-term outcomes.

## Contributors

AIT, BH, CC, SC and CRL conceptualized the study and participated to funding acquisition AIT, AA, SK overviewed study methodology. PBL, EL and LR performed data curation and formal analysis. PBL and CRL had direct access to the data and verified the data. PBL performed data visualization and wrote the original draft. AIT, CC, and CRL supervised the study and project administration. PBL, AIT, BH, CC, SC, TP, AL, AA, JN, GG, DM, BS, BL and CRL participated in manuscript writing, reviewing and editing.

All authors confirm that they had full access to all the data in the study and accept responsibility to submit for publication.

## Data sharing statement

The access to data from the SNDS database is restricted. We are not allowed to extract raw data from the database and will therefore not be able to share the raw data from this study. We will share the data extraction scripts upon request to researchers with SNDS granted access.

## AI use statement

ChatGPT 4 was used to improve readability and language of the text with oversight of the authors.

## Declaration of interests

PBL, BL, CC, BS, LRS, ELU, SK, GG, AAX, AT, BH and CRL received support from INSERM and the French Ministry of Health (Messidore 2022 no. 97 - Innovative methodologies, devices, tools and research in clinical trials using health data and biobanks) as reported in the funding section. SC holds shares in ALIFERT, JELLYNOV companies and received personal fees from Bariatek, Novonordisk, Eli Lilly, Pfizer, Fresenius Kabi, Ipsen Pharma, Janssen-Cilag, Boehringer Ingelheim and Novartis. AL received payments from Johnson & Johnson, Medtronic and Gore for the organization of surgical workshop for bariatric surgeons and support from Novo Nordisk for attending congress. LR, TP, JN have nothing to declare.

## Appendix ASupplementary data

Supplementary methods, Figs. S1 and S2, Tables S1–S5

French abstract
