# Supplementary material for: Maternal and neonatal outcomes of pregnancies after metabolic bariatric surgery: a retrospective population-based study
Source: Lancet Reg Health Eur. 2025 Mar 22;51:101263. doi: 10.1016/j.lanepe.2025.101263 (PMC11982497; doi:10.1016/j.lanepe.2025.101263)
Supplement: Supplementary methods, Figs. S1 and S2, Tables S1–S5 [file mmc1.docx]

Supplementary appendix

[Supplementary methods 2](#_Toc189301239)

[1 Bariatric surgery (BS) identification 2](#_Toc189301240)

[2 Mother to child linkage 2](#_Toc189301241)

[3 Mother variables 2](#_Toc189301242)

[2.1 Mother comorbidities/characteristics at pregnancy 2](#_Toc189301245)

[2.2 Imputation of post-BS mothers obesity status at pregnancy 2](#_Toc189301246)

[2.3 Specific post-BS variables 3](#_Toc189301247)

[4 Outcomes definition 3](#_Toc189301248)

[3.1 Gestational diabetes 3](#_Toc189301250)

[3.2 Gestational hypertension 4](#_Toc189301251)

[3.3 Preeclampsia or eclampsia 4](#_Toc189301252)

[3.4 Small for gestational age neonates (SGA) and large for gestational age neonates (LGA) 4](#_Toc189301253)

[3.5 Termination of pregnancy 4](#_Toc189301254)

[3.6 Prematurity 4](#_Toc189301255)

[3.7 Stillbirth 4](#_Toc189301256)

[3.8 Perinatal death 4](#_Toc189301257)

[Counterfactual analysis 4](#_Toc189301258)

[Supplementary tables 6](#_Toc189301259)

[1 Supplementary table 1: Adverse pregnancy and neonatal outcomes in post-BS vs. control according to BS type 6](#_Toc189301260)

[2 Supplementary Table 2: Adverse pregnancy and neonatal outcomes in post-BS vs. control according to time from BS to pregnancy 6](#_Toc189301261)

[3 Supplementary Table 3: Adverse pregnancy and neonatal outcomes in post-BS vs. control according to pre-BS obesity class 9](#_Toc189301262)

[4 Supplementary Table 4: Adverse pregnancy and neonatal outcomes in post-BS vs. control according to malnutrition status (from BS to delivery) 11](#_Toc189301263)

[5 Supplementary table 5: Characteristics of the pre-BS Pregnancy 2 vs pregnancy 1 population (counterfactual analysis) 12](#_Toc189301264)

[Supplementary figures 14](#_Toc189301265)

[1 Supplementary Figure 1: Standardized difference of matching variables before and after matching 14](#_Toc189301266)

[2 Supplementary Figure 2: Adverse pregnancy and neonatal outcomes in pre-BS pregnancies #2 vs. #1 (conterfactual analysis) 14](#_Toc189301267)

[References 16](#_Toc189301268)

# Supplementary methods

## Bariatric surgery (BS) identification

BS were identified using procedure codes from the French National Procedures Classification (HFFC018/HFFA011/HFMC006/HFMA010 for sleeve gastrectomy, HFCC003/HFCA001 for gastric bypass, HFFC004/HFFA001 for bilio-pancreatic derivation and HFMC007/HFMA009 for gastric banding) associated with an obesity diagnoses (E66 ICD-10 code).

## Mother to child linkage

Mother to child linkage was performed using the linkage variable NIR_ANO_MAM available from 2013 to 2018 and ID_MAM_ENF available from 2019 onwards as described ^1^. To avoid mislinkage, further verifications were applied before linkage:

- Delivery hospital admission of the mother in the same hospital than the birth admission stay of the child
- Mother delivery date (date of the delivery act when specified and the date of hospital admission for delivery when unspecified) = child birth date ± 2 days

## Mother variables


### Mother comorbidities/characteristics at pregnancy

- **Nulliparous status**. Prior hospital admission for birth were looked in all cases from 2005 (maximum depth of available SNDS data). Mothers were considered as nulliparous if no prior birth was detected and if the birth stay ICD-10 coding did not involve codes referring to previous parities.
- **Medically assisted procreation** was detected in SNDS using a previously defined algorithm as ≥ 1 infertility drug dispensation and or medically assisted procreation act in the 12 months preceding pregnancy conception date^2^.
- **Treated type 1 diabetes** was defined following a previously defined algorithm adapted for post-BS context ^3^. Treated type 1 diabetes was considered if all the following criteria were verified
  - ≥ 1 insulin dispensation in the 6 months before pregnancy conception date
  - no other anti-diabetes treatment in the 12 months preceding pregnancy conception date
  - ≥ 1insulin dispensation in the 6-12 months post-birth period.
- **Treated type 2 diabetes** was defined following a previously defined algorithm adapted for post-BS context ^3^. Treated type 2 diabetes was considered if the following criteria were verified
  - ≥ 1 non-insulin anti diabetes drug dispensation in the 6 months preceding pregnancy conception date

OR

- - ≥ 1 insulin dispensation in the 6 months before pregnancy conception AND ≥ 1 non-insulin anti diabetes drug dispensation in the 6-12 months post birth

AND

- - no type 1 diabetes
- **Treated hypertension** was defined as ≥ 1 anti-hypertension drug dispensation in the 6 months preceding pregnancy conception date
- **Treated sleep apnea syndrome** was defined as ≥ 1 reimbursement of a service for sleep apnea machine in the 12 months preceding pregnancy conception date
- **Social deprivation index** was obtained from the SNDS cartography of diseases and represents the quintiles socio-economic status of the commune of residency of the person (1 being the most socially advantaged and 5 the most disadvantaged)
- **Health coverage assistance** was obtained from the SNDS cartography of diseases and represents the fact the person is receiving state financial support for her health coverage expenses. Specifically we looked at *couverture medicale universelle* (CMU) until 2019 and *complementaire social solidaire* (C2S) from 2019

### Imputation of post-BS mothers obesity status at pregnancy

#### Imputation of obesity status (BMI ≥ 30 kg/m^2^)

Pre-BS BMI class was collected using ICD-10 codes (exhaustive in the context of BS) that allowed categorization for obesity (BMI ≥ 30 kg/m^2^) and severe obesity (BMI ≥ 40 kg/m^2^). BMI class at pregnancy was collected using ICD-10 associated codes of the birth hospital admission (probably less exhaustive in the context of pregnancy). Indeed, no coding could either mean no obesity or that the obesity diagnostic was not coded because of non-exhaustive coding in that maternity. We then applied the following steps to impute obesity status at pregnancy:

- **Learning dataset.** We considered a maternity exhaustive for obesity coding if their obesity prevalence in post-BS women was ≥ 35% which is consistent with post-BS obesity prevalence in the context of pregnant women in the literature ^4^. This subset contained 4525 pregnancies and was split into a train dataset (80% of data) and test data set (20%). We used an oversampling method, specifically SMOTE (Synthetic Minority Oversampling Technique), to rebalance the two classes (obesity vs. no-obesity).
- **Variable selection.** Predictive variables were selected based on literature knowledge about post-BS weight loss prediction. The selected variables were age, BMI class at BS, time from BS to pregnancy, BS type (sleeve, gastric bypass, banding or bilio-pancreatic derivation), multiple surgery status (primary or revisional), comorbidities status at BS and at pregnancy and their interactions (diabetes, hypertension, sleep apnea syndrome), socio economic status, GLPR agonists use, insulin use, antidepressants use.
- **Random forest model construction and performance.** We optimized hyper parameters using caret r package with 4 fold cross validation and trained a random forest model on the training dataset. The metric to optimize was set to specificity in order to avoid having too many post-BS pregnancies imputed as with obesity when it is not the case. The performance of the model (on the test dataset) was 72% accuracy, with a high specificity of 86% and a sensibility of 41%.
- **Obesity imputation.** To complete missing obesity class at pregnancy in post-BS women, we applied our model all women who didn’t have an obesity coding at pregnancy. Before imputation, 8922 (16.0%) post-BS women were tagged with obesity at pregnancy. After imputation, this number increased to 14996 (26.8%).
- **Variable importance.** The most important variables for BMI prediction (calculated using permutation method) were BMI before BS, Type 2 diabetes status, age and time from BS to pregnancy.

#### Imputation of severe obesity status (BMI ≥ 40 kg/m^2^)

Pre-BS severe obesity status using a similar approach:

- **Learning dataset.** We selected all pregnancies from post-BS women with an obesity ICD-10 code at the birth hospital admission. This subset contained 9394 pregnancies and was split into a train dataset (80% of data) and test data set (20%)
- **Variable selection.** Predictive variables were identical as for obesity imputation
- **Random forest model construction and performance.** Model construction method was the same as for obesity imputation except that the metric chosen was accuracy. The performance of the model (on the test dataset) was 75% accuracy, with a high specificity of 84% and a 37% sensitivity.
- **Obesity imputation.** To complete missing severe obesity status at pregnancy in post-BS women, we applied our model on all women with obesity status (including imputed) at pregnancy. Before imputation, 1920 (3.4%) post-BS women were tagged with severe obesity at pregnancy. After imputation, this number increased to 4207 (7.5 %).
- **Variable importance.** The most important variables for severe obesity prediction (calculated using permutation method) were BMI before BS, age,time from BS to pregnancy and multiple surgery status.

### Specific post-BS variables

- **Surgery to pregnancy interval** was defined as the delay between surgery and pregnancy conception date and was categorized into < 6 months, 6-12 months, 1-2 years, 2-5 years and ≥ 5 years
- **Malnutrition from BS to pregnancy** was defined by the presence of at least one of the following criteria from BS date to birth:
  - Hospital ICD-10 diagnoses code for malnutrition or severe eating disorder
  - Hospital CCAM act of administration of artificial nutrition (enteral or parenteral)
  - Ambulatory forfeit reimbursement for the home administration of artificial nutrition (enteral or parenteral)

## Outcomes definition


### Gestational diabetes

Gestational diabetes was identified using an adaptation (to include post-BS specificities) of a previously defined and used algorithm ^3^. Briefly, gestational diabetes was defined if **at least one of the following criteria** was met:

- diagnosis of diabetes recorded from pregnancy date to delivery date (ICD-10 codes E10–E14, O240–O244, O249)
- insulin dispensed at least once during pregnancy
- at least 200 glucose strips dispensed during pregnancy on at least 2 different occasions

without :

- previous or subsequent diagnoses of diabetes
- diabetes medication dispensation in the 6 month preceding pregnancy

### Gestational hypertension

Gestational hypertension was identified using an adaptation (to include post-BS specificities) of a previously defined and used algorithm^5^. Briefly, gestational hypertension was defined if the following criteria was met:

- diagnosis of gestational hypertension recorded from pregnancy date to delivery date (ICD-10 code O13)

without:

- preexisting chronic diagnoses of hypertension (see details further)
- dispensation of anti-hypertension medication in the 6 months preceding pregnancy

### Preeclampsia or eclampsia

Preeclampsia or eclampsia was defined if a diagnoses of preeclampsia/eclampsia (ICD codes O14 and O15) was recorded from pregnancy date to delivery date as previously defined ^5^.

### Small for gestational age neonates (SGA) and large for gestational age neonates (LGA)

For each child, we estimated the growth percentile at birth using Audipog reference curves giving growth percentiles according to the gestational age based on 204 316 children born in France between 1999 and 2005 depending on age, sex, weight but not on race and ethnicity^[[1]](#footnote-1)^. SGA was defined as having a growth percentile below 10% and LGA was defined as having a growth percentile above 90%.

### Termination of pregnancy

Termination of pregnancy was defined by a term > 22 weeks after the last menstrual period and the presence of at least one of the following criteria :

- Gravid uterus evacuation by aspiration/medication codes JNJD001/JNJD002/JNJP001
- ICD-10 codes indicating termination of pregnancy (Z3711, Z3731,Z3741,Z3761, Z3771)
- ICD-10 codes indicating abortion (Z640, O04, O05, O06)

### Prematurity

Prematurity was defined as live or still birth before 37 weeks after the last menstrual period (LMP) and subsequently categorized into

- Moderate prematurity 32-37 weeks after LMP
- Severe prematurity 28-32 weeks after LMP
- Extreme prematurity < 28 weeks after LMP

### Stillbirth

Stillbirth was defined by a term > 22 weeks after the last menstrual period and the presence of at least one of the following criteria :

- ICD-10 codes indicating stillbirth in the delivery stay of the mother (Z3710, Z3730, Z3740, Z3760, Z3770)
- ICD-10 codes indicating stillbirth in the birth stay of the child (P95)

### Perinatal death

Perinatal death was defined as a death date within the 7 days following the birth date of the child. Death date was obtained by matching the unique id of the child with the IR_BEN_R table of the SNDS.

# Counterfactual analysis

The purpose of this analysis was to identify outcomes potentially confounded by age and parity changes from pre-BS to post-BS pregnancies. Among the pre-BS pregnancies population, we identified women with at least two pregnancies before BS. For the counterfactual analysis, the furthest pregnancy from BS (Pregnancy #1) and the closest to BS (Pregnancy #2) were selected with a minimum of two years between pregnancies in order to be the closest to the post- vs. pre-BS analysis.

Among women with pre-BS pregnancies, 10,694 women had two pregnancies before BS and were included in the counterfactual study. Age was higher for pre-BS pregnancy#2 than for pre-BS pregnancy#1 (30.1 ± 4.7 vs. 26.5 ± 4.7 years; p<0.01).

Pre-BS pregnancies characteristics (counterfactual population) are shown in Supplementary Table 5.

Results are shown in supplementary figure 1. Most of the associations found in post-BS vs. pre-BS pregnancy in the same mother were not found in the same direction in the counterfactual analysis of pre-BS pregnancy #2 vs. pre-BS pregnancy#1 at the exception of gestational hypertension and preeclampsia. When comparing post-BS with pre-BS pregnancies in the same woman, the ORs were 0.16 for gestational hypertension and 0.17 for preeclampsia. However, the ORs found in the counterfactual analysis, respectively 0.75 and 0.68, do not fully account for the protective associations observed in post-BS pregnancies. This suggests that part, but not all, of the risk reductions observed in post-BS vs. pre-BS pregnancy for these outcomes could be attributed to the higher frequency of primiparity in pre-BS pregnancies, which is a known risk factor for gestational hypertension and preeclampsia^18,19^.

Overall, we conclude from the counterfactual analysis that the observed associations in the post-BS pregnancies compared with pre-BS pregnancies were not explained by the greater age and parity of the mother at the time of post-BS pregnancy.

# Supplementary tables

## Supplementary table 1: Adverse pregnancy and neonatal outcomes in post-BS vs. control according to BS type

| Outcome | Subgroup | OR | IC inf | IC sup | p value | Post-BS, n (%) | Control, n (%) |  |  | Difference (%) | Interaction p value |
| --- | --- | --- | --- | --- | --- | --- | --- | --- | --- | --- | --- |
| Perinatal death (<7 days) | Sleeve | 1.43 | 1.01 | 2.03 | 0.046 | 45 (0.12) | 114 (0.07) |  |  | +0.04 | ref |
| Perinatal death (<7 days) | Gastric bypass | 1.73 | 1.03 | 2.90 | 0.037 | 21 (0.16) | 48 (0.09) |  |  | +0.07 | 0.752 |
| Perinatal death (<7 days) | Gastric band | 1.67 | 0.50 | 5.62 | 0.688 | 5 (0.11) | 12 (0.07) |  |  | +0.04 | 0.897 |
| Stillbirth | Sleeve | 1.19 | 1.03 | 1.37 | 0.016 | 278 (0.73) | 872 (0.57) |  |  | +0.16 | ref |
| Stillbirth | Gastric bypass | 1.39 | 1.09 | 1.77 | 0.007 | 95 (0.72) | 277 (0.53) |  |  | +0.19 | 0.710 |
| Stillbirth | Gastric band | 1.05 | 0.66 | 1.68 | 0.842 | 23 (0.52) | 83 (0.47) |  |  | +0.05 | 0.393 |
| Prematurity (<37W) | Sleeve | 1.23 | 1.17 | 1.28 | <0.0001 | 3060 (8.11) | 10129 (6.7) |  |  | +1.41 | ref |
| Prematurity (<37W) | Gastric bypass | 1.46 | 1.36 | 1.57 | <0.0001 | 1235 (9.48) | 3480 (6.66) |  |  | +2.82 | 0.0003 |
| Prematurity (<37W) | Gastric band | 1.04 | 0.90 | 1.20 | 0.805 | 319 (7.23) | 1221 (6.91) |  |  | +0.31 | 0.030 |
| Small-for-gestational-age | Sleeve | 1.74 | 1.68 | 1.81 | <0.0001 | 5071 (13.44) | 12484 (8.26) |  |  | +5.18 | ref |
| Small-for-gestational-age | Gastric bypass | 1.88 | 1.77 | 2.00 | <0.0001 | 1821 (13.98) | 4187 (8.01) |  |  | +5.96 | 0.027 |
| Small-for-gestational-age | Gastric band | 1.19 | 1.02 | 1.38 | 0.023 | 410 (9.29) | 1403 (7.94) |  |  | +1.35 | <0.0001 |
| Large-for-gestational-age | Sleeve | 0.47 | 0.44 | 0.49 | <0.0001 | 2035 (5.66) | 16181 (11.23) |  |  | -5.57 | ref |
| Large-for-gestational-age | Gastric bypass | 0.39 | 0.36 | 0.43 | <0.0001 | 613 (4.92) | 5687 (11.43) |  |  | -6.51 | 0.002 |
| Large-for-gestational-age | Gastric band | 0.96 | 0.84 | 1.08 | 0.688 | 456 (10.92) | 1910 (11.4) |  |  | -0.48 | <0.0001 |
| Gestational diabetes | Sleeve | 0.57 | 0.54 | 0.59 | <0.0001 | 4451 (11.95) | 28598 (18.72) |  |  | -6.78 | ref |
| Gestational diabetes | Gastric bypass | 0.66 | 0.62 | 0.71 | <0.0001 | 1828 (14.21) | 10253 (19.44) |  |  | -5.24 | 0.0003 |
| Gestational diabetes | Gastric band | 1.20 | 1.06 | 1.37 | 0.001 | 941 (21.61) | 3304 (18.54) |  |  | +3.07 | <0.0001 |
| Preeclampsia | Sleeve | 0.58 | 0.53 | 0.63 | <0.0001 | 637 (1.67) | 4246 (2.78) |  |  | -1.11 | ref |
| Preeclampsia | Gastric bypass | 0.47 | 0.40 | 0.56 | <0.0001 | 178 (1.35) | 1460 (2.77) |  |  | -1.42 | 0.042 |
| Preeclampsia | Gastric band | 1.03 | 0.84 | 1.26 | 0.842 | 137 (3.08) | 536 (3.01) |  |  | +0.07 | <0.0001 |
| Gestational hypertension | Sleeve | 0.56 | 0.51 | 0.62 | <0.0001 | 516 (1.35) | 3640 (2.38) |  |  | -1.03 | ref |
| Gestational hypertension | Gastric bypass | 0.42 | 0.35 | 0.50 | <0.0001 | 142 (1.08) | 1343 (2.55) |  |  | -1.47 | 0.014 |
| Gestational hypertension | Gastric band | 1.10 | 0.87 | 1.40 | 0.688 | 128 (2.87) | 478 (2.68) |  |  | +0.19 | 0.000 |

Odds ratios are calculated from generalized estimated equations adjusted for age (continuous), health coverage assistance, deprivation index quintiles, pregnancy obesity status, sleep apnea syndrome and medically assisted reproduction for that pregnancy. Confidence intervals are enlarged proportionally to the p-value increase after multiple comparison adjustment by using the false discovery rate method. Interactions for each subgroup were tested with sleeve gastrectomy as a reference. False discovery rate multiple comparison adjustment was applied for calculating p_interaction_ values. BS: metabolic bariatric surgery; W: weeks after last menstrual period; ref: reference

## Supplementary Table 2: Adverse pregnancy and neonatal outcomes in post-BS vs. control according to time from BS to pregnancy

| Outcome | Subgroup | OR | IC inf | IC sup | p value | Post-BS (%) | Control (%) |  |  | Difference (%) | Interaction p value |
| --- | --- | --- | --- | --- | --- | --- | --- | --- | --- | --- | --- |
| Perinatal death (<7 days) | 0-6 months | 1.81 | 0.62 | 5.31 | 0.303 | 5 (0.17) | 12 (0.1) |  |  | +0.07 | 0.603 |
| Perinatal death (<7 days) | 6-12 months | 3.37 | 1.55 | 7.34 | 0.002 | 14 (0.24) | 16 (0.07) |  |  | +0.17 | 0.018 |
| Perinatal death (<7 days) | 1-2 years | 1.65 | 0.94 | 2.89 | 0.082 | 19 (0.14) | 44 (0.08) |  |  | +0.06 | 0.403 |
| Perinatal death (<7 days) | 2-5 years | 1.10 | 0.69 | 1.73 | 0.697 | 25 (0.1) | 79 (0.08) |  |  | +0.02 | ref |
| Perinatal death (<7 days) | >5 years | 1.20 | 0.53 | 2.71 | 0.661 | 8 (0.09) | 24 (0.07) |  |  | +0.02 | 0.983 |
| Stillbirth | 0-6 months | 1.31 | 0.74 | 2.29 | 0.35 | 17 (0.59) | 54 (0.47) |  |  | +0.12 | 0.979 |
| Stillbirth | 6-12 months | 1.10 | 0.77 | 1.58 | 0.597 | 41 (0.7) | 145 (0.62) |  |  | +0.08 | 0.884 |
| Stillbirth | 1-2 years | 1.14 | 0.88 | 1.47 | 0.314 | 88 (0.65) | 283 (0.52) |  |  | +0.13 | 0.797 |
| Stillbirth | 2-5 years | 1.14 | 0.95 | 1.37 | 0.163 | 170 (0.69) | 549 (0.55) |  |  | +0.13 | ref |
| Stillbirth | >5 years | 1.50 | 1.13 | 1.98 | 0.004 | 80 (0.9) | 202 (0.57) |  |  | +0.33 | 0.329 |
| Prematurity (<37W) | 0-6 months | 1.17 | 0.99 | 1.38 | 0.065 | 239 (8.36) | 850 (7.39) |  |  | +0.97 | 0.788 |
| Prematurity (<37W) | 6-12 months | 1.22 | 1.08 | 1.37 | 0.001 | 475 (8.18) | 1601 (6.89) |  |  | +1.29 | 0.884 |
| Prematurity (<37W) | 1-2 years | 1.35 | 1.25 | 1.46 | <0.0001 | 1146 (8.59) | 3573 (6.68) |  |  | +1.91 | 0.190 |
| Prematurity (<37W) | 2-5 years | 1.21 | 1.15 | 1.29 | <0.0001 | 1976 (8.07) | 6504 (6.63) |  |  | +1.44 | ref |
| Prematurity (<37W) | >5 years | 1.37 | 1.24 | 1.51 | <0.0001 | 790 (9.03) | 2335 (6.64) |  |  | +2.39 | 0.115 |
| Small-for-gestational-age | 0-6 months | 1.95 | 1.72 | 2.21 | <0.0001 | 436 (15.25) | 976 (8.49) |  |  | +6.76 | 0.011 |
| Small-for-gestational-age | 6-12 months | 1.86 | 1.70 | 2.04 | <0.0001 | 831 (14.31) | 1921 (8.27) |  |  | +6.04 | 0.018 |
| Small-for-gestational-age | 1-2 years | 1.74 | 1.63 | 1.84 | <0.0001 | 1814 (13.59) | 4441 (8.3) |  |  | +5.29 | 0.190 |
| Small-for-gestational-age | 2-5 years | 1.66 | 1.58 | 1.73 | <0.0001 | 3137 (12.8) | 7972 (8.13) |  |  | +4.68 | ref |
| Small-for-gestational-age | >5 years | 1.67 | 1.55 | 1.80 | <0.0001 | 1101 (12.58) | 2791 (7.93) |  |  | +4.65 | 0.983 |
| Large-for-gestational-age | 0-6 months | 0.40 | 0.33 | 0.48 | <0.0001 | 145 (5.33) | 1274 (11.76) |  |  | -6.43 | 0.022 |
| Large-for-gestational-age | 6-12 months | 0.35 | 0.30 | 0.41 | <0.0001 | 239 (4.34) | 2434 (11.01) |  |  | -6.67 | <0.0001 |
| Large-for-gestational-age | 1-2 years | 0.45 | 0.42 | 0.50 | <0.0001 | 688 (5.41) | 5548 (10.9) |  |  | -5.48 | 0.113 |
| Large-for-gestational-age | 2-5 years | 0.50 | 0.48 | 0.54 | <0.0001 | 1413 (6.04) | 10510 (11.25) |  |  | -5.2 | ref |
| Large-for-gestational-age | >5 years | 0.58 | 0.53 | 0.63 | <0.0001 | 623 (7.42) | 4059 (12.06) |  |  | -4.64 | 0.006 |
| Gestational diabetes | 0-6 months | 0.56 | 0.46 | 0.67 | <0.0001 | 327 (11.3) | 2018 (17.38) |  |  | -6.08 | 0.573 |
| Gestational diabetes | 6-12 months | 0.54 | 0.49 | 0.60 | <0.0001 | 567 (9.76) | 3828 (16.31) |  |  | -6.55 | 0.023 |
| Gestational diabetes | 1-2 years | 0.58 | 0.54 | 0.62 | <0.0001 | 1431 (10.79) | 9100 (16.86) |  |  | -6.07 | 0.113 |
| Gestational diabetes | 2-5 years | 0.64 | 0.61 | 0.67 | <0.0001 | 3330 (13.86) | 19200 (19.37) |  |  | -5.51 | ref |
| Gestational diabetes | >5 years | 0.75 | 0.70 | 0.80 | <0.0001 | 1577 (18.39) | 8097 (22.79) |  |  | -4.4 | 0.004 |
| Preeclampsia | 0-6 months | 0.49 | 0.34 | 0.70 | <0.0001 | 41 (1.41) | 353 (3.04) |  |  | -1.63 | 0.109 |
| Preeclampsia | 6-12 months | 0.45 | 0.33 | 0.60 | <0.0001 | 76 (1.3) | 660 (2.81) |  |  | -1.52 | 0.018 |
| Preeclampsia | 1-2 years | 0.49 | 0.42 | 0.58 | <0.0001 | 179 (1.33) | 1416 (2.62) |  |  | -1.3 | 0.098 |
| Preeclampsia | 2-5 years | 0.61 | 0.55 | 0.68 | <0.0001 | 439 (1.77) | 2802 (2.83) |  |  | -1.06 | ref |
| Preeclampsia | >5 years | 0.82 | 0.70 | 0.97 | 0.016 | 217 (2.44) | 1026 (2.89) |  |  | -0.45 | 0.004 |
| Gestational hypertension | 0-6 months | 0.42 | 0.28 | 0.65 | <0.0001 | 37 (1.27) | 340 (2.93) |  |  | -1.65 | 0.109 |
| Gestational hypertension | 6-12 months | 0.42 | 0.31 | 0.58 | <0.0001 | 58 (0.99) | 563 (2.4) |  |  | -1.41 | 0.023 |
| Gestational hypertension | 1-2 years | 0.53 | 0.45 | 0.64 | <0.0001 | 174 (1.29) | 1275 (2.36) |  |  | -1.07 | 0.403 |
| Gestational hypertension | 2-5 years | 0.60 | 0.54 | 0.68 | <0.0001 | 356 (1.44) | 2376 (2.4) |  |  | -0.96 | ref |
| Gestational hypertension | >5 years | 0.69 | 0.57 | 0.84 | <0.0001 | 164 (1.85) | 918 (2.58) |  |  | -0.74 | 0.362 |

Odds ratios are calculated from generalized estimated equations adjusted for age (continuous), health coverage assistance, deprivation index quintiles, pregnancy obesity status, sleep apnea syndrome and medically assisted reproduction for that pregnancy. Confidence intervals are enlarged proportionally to the p-value increase after multiple comparison adjustment by using the false discovery rate method. Interactions for each subgroup were tested with 2-5 years as a reference. False discovery rate multiple comparison adjustment was applied for calculating p_interaction_ values. BS: metabolic bariatric surgery; W: weeks after last menstrual period; ref: reference

## Supplementary Table 3: Adverse pregnancy and neonatal outcomes in post-BS vs. control according to pre-BS obesity class

| Outcome | Pre-BS obesity class | OR | IC inf | IC sup | p value | Post-BS (%) | Control (%) |  |  | Difference (%) | Interaction p value |
| --- | --- | --- | --- | --- | --- | --- | --- | --- | --- | --- | --- |
| Perinatal death (<7 days) | 30-40 kg/m2 | 1.61 | 0.95 | 2.73 | 0.077 | 20 (0.11) | 47 (0.07) |  |  | +0.05 | 0.984 |
| Perinatal death (<7 days) | 40-50 kg/m2 | 1.65 | 1.16 | 2.35 | 0.005 | 47 (0.14) | 108 (0.08) |  |  | +0.06 | ref |
| Perinatal death (<7 days) | >=50 kg/m2 | 0.63 | 0.22 | 1.82 | 0.435 | 4 (0.08) | 20 (0.1) |  |  | -0.02 | 0.172 |
| Stillbirth | 30-40 kg/m2 | 1.27 | 1.02 | 1.58 | 0.03 | 115 (0.65) | 350 (0.49) |  |  | +0.16 | 0.984 |
| Stillbirth | 40-50 kg/m2 | 1.23 | 1.06 | 1.42 | 0.008 | 241 (0.72) | 738 (0.55) |  |  | +0.17 | ref |
| Stillbirth | >=50 kg/m2 | 1.02 | 0.70 | 1.48 | 0.923 | 40 (0.8) | 145 (0.73) |  |  | +0.07 | 0.328 |
| Prematurity (<37W) | 30-40 kg/m2 | 1.40 | 1.31 | 1.49 | <0.0001 | 1530 (8.74) | 4432 (6.32) |  |  | +2.42 | 0.065 |
| Prematurity (<37W) | 40-50 kg/m2 | 1.28 | 1.23 | 1.35 | <0.0001 | 2724 (8.3) | 8719 (6.62) |  |  | +1.67 | ref |
| Prematurity (<37W) | >=50 kg/m2 | 0.86 | 0.76 | 0.99 | 0.028 | 372 (7.56) | 1712 (8.69) |  |  | -1.13 | <0.0001 |
| Small-for-gestational-age | 30-40 kg/m2 | 1.63 | 1.55 | 1.72 | <0.0001 | 2271 (12.97) | 5888 (8.4) |  |  | +4.57 | 0.113 |
| Small-for-gestational-age | 40-50 kg/m2 | 1.74 | 1.68 | 1.81 | <0.0001 | 4371 (13.31) | 10734 (8.15) |  |  | +5.16 | ref |
| Small-for-gestational-age | >=50 kg/m2 | 2.05 | 1.84 | 2.28 | <0.0001 | 677 (13.75) | 1479 (7.5) |  |  | +6.25 | 0.019 |
| Large-for-gestational-age | 30-40 kg/m2 | 0.55 | 0.51 | 0.59 | <0.0001 | 1009 (6.06) | 6893 (10.34) |  |  | -4.28 | 0.072 |
| Large-for-gestational-age | 40-50 kg/m2 | 0.50 | 0.47 | 0.52 | <0.0001 | 1800 (5.74) | 13822 (11.01) |  |  | -5.27 | ref |
| Large-for-gestational-age | >=50 kg/m2 | 0.33 | 0.28 | 0.37 | <0.0001 | 299 (6.35) | 3110 (16.53) |  |  | -10.18 | <0.0001 |
| Gestational diabetes | 30-40 kg/m2 | 0.80 | 0.75 | 0.85 | <0.0001 | 2173 (12.59) | 10873 (15.36) |  |  | -2.77 | 0.046 |
| Gestational diabetes | 40-50 kg/m2 | 0.72 | 0.69 | 0.75 | <0.0001 | 4311 (13.28) | 23658 (17.79) |  |  | -4.51 | ref |
| Gestational diabetes | >=50 kg/m2 | 0.29 | 0.27 | 0.32 | <0.0001 | 748 (15.38) | 7712 (38.71) |  |  | -23.33 | <0.0001 |
| Preeclampsia | 30-40 kg/m2 | 0.76 | 0.65 | 0.87 | <0.0001 | 296 (1.67) | 1573 (2.22) |  |  | -0.55 | 0.129 |
| Preeclampsia | 40-50 kg/m2 | 0.65 | 0.59 | 0.72 | <0.0001 | 553 (1.66) | 3466 (2.61) |  |  | -0.94 | ref |
| Preeclampsia | >=50 kg/m2 | 0.31 | 0.24 | 0.38 | <0.0001 | 103 (2.07) | 1218 (6.11) |  |  | -4.05 | <0.0001 |
| Gestational hypertension | 30-40 kg/m2 | 0.76 | 0.65 | 0.88 | 0.0002 | 244 (1.38) | 1301 (1.84) |  |  | -0.46 | 0.065 |
| Gestational hypertension | 40-50 kg/m2 | 0.62 | 0.55 | 0.68 | <0.0001 | 447 (1.34) | 3031 (2.28) |  |  | -0.93 | ref |
| Gestational hypertension | >=50 kg/m2 | 0.34 | 0.27 | 0.43 | <0.0001 | 98 (1.97) | 1140 (5.72) |  |  | -3.76 | <0.0001 |

Odds ratios are calculated from generalized estimated equations adjusted for age (continuous), health coverage assistance, deprivation index quintiles, pregnancy obesity status, sleep apnea syndrome and medically assisted reproduction for that pregnancy. Confidence intervals are enlarged proportionally to the p-value increase after multiple comparison adjustment by using the false discovery rate method. Interactions for each subgroup were tested with BMI 40-50 kg/m2 as a reference. False discovery rate multiple comparison adjustment was applied for calculating pinteraction values. BS: metabolic bariatric surgery; W: weeks after last menstrual period; ref: reference

## Supplementary Table 4: Adverse pregnancy and neonatal outcomes in post-BS vs. control according to malnutrition status (from BS to delivery)

| Outcome | Malnutrition status | OR | IC inf | IC sup | p value | Post-BS (%) | Control (%) | Difference (%) | Interaction p value |
| --- | --- | --- | --- | --- | --- | --- | --- | --- | --- |
| Perinatal death (<7 days) | Malnutrition | 2.29 | 0.42 | 12.51 | 0.338 | 3 (0.25) | 4 (0.08) | +0.17 | 0.555 |
| Perinatal death (<7 days) | No malnutrition | 1.49 | 1.12 | 1.98 | 0.006 | 68 (0.12) | 171 (0.08) | +0.05 | ref |
| Stillbirth | Malnutrition | 1.50 | 0.66 | 3.40 | 0.338 | 8 (0.67) | 24 (0.5) | +0.17 | 0.888 |
| Stillbirth | No malnutrition | 1.19 | 1.06 | 1.35 | 0.004 | 388 (0.71) | 1209 (0.55) | +0.16 | ref |
| Prematurity (<37W) | Malnutrition | 2.45 | 1.99 | 3.00 | <0.0001 | 180 (15.33) | 322 (6.83) | +8.5 | <0.0001 |
| Prematurity (<37W) | No malnutrition | 1.24 | 1.20 | 1.29 | <0.0001 | 4446 (8.22) | 14543 (6.71) | +1.51 | ref |
| Small-for-gestational-age | Malnutrition | 2.38 | 1.96 | 2.88 | <0.0001 | 199 (16.95) | 376 (7.97) | +8.98 | 0.003 |
| Small-for-gestational-age | No malnutrition | 1.72 | 1.67 | 1.78 | <0.0001 | 7120 (13.16) | 17727 (8.18) | +4.99 | ref |
| Large-for-gestational-age | Malnutrition | 0.40 | 0.29 | 0.57 | <0.0001 | 59 (5.25) | 542 (12.09) | -6.84 | 0.335 |
| Large-for-gestational-age | No malnutrition | 0.48 | 0.46 | 0.50 | <0.0001 | 3049 (5.91) | 23289 (11.28) | -5.37 | ref |
| Gestational diabetes | Malnutrition | 0.73 | 0.58 | 0.91 | 0.003 | 201 (17.51) | 1035 (21.76) | -4.25 | 0.335 |
| Gestational diabetes | No malnutrition | 0.63 | 0.61 | 0.65 | <0.0001 | 7031 (13.16) | 41212 (18.82) | -5.66 | ref |
| Preeclampsia | Malnutrition | 0.69 | 0.43 | 1.11 | 0.142 | 26 (2.18) | 151 (3.17) | -0.99 | 0.648 |
| Preeclampsia | No malnutrition | 0.59 | 0.55 | 0.64 | <0.0001 | 926 (1.69) | 6107 (2.79) | -1.1 | ref |
| Gestational hypertension | Malnutrition | 0.64 | 0.39 | 1.06 | 0.094 | 22 (1.85) | 142 (2.99) | -1.14 | 0.888 |
| Gestational hypertension | No malnutrition | 0.57 | 0.53 | 0.62 | <0.001 | 767 (1.4) | 5330 (2.43) | -1.03 | ref |

Odds ratios are calculated from generalized estimated equations adjusted for age (continuous), health coverage assistance, deprivation index quintiles, pregnancy obesity status, sleep apnea syndrome and medically assisted reproduction for that pregnancy. Confidence intervals are enlarged proportionally to the p-value increase after multiple comparison adjustment by using the false discovery rate method. Interactions for each subgroup were tested with no malnutrition as a reference. False discovery rate multiple comparison adjustment was applied for calculating pinteraction values. BS: metabolic bariatric surgery; W: weeks after last menstrual period; ref: reference

## Supplementary table 5: Characteristics of the pre-BS Pregnancy 2 vs pregnancy 1 population (counterfactual analysis)

| **Characteristics** | **Pre-BS pregnancy #2 vs. Pre-BS pregnancy #1** | | |
| --- | --- | --- | --- |
|  | Pre-BS pregnancy #2 | Pre-BS pregnancy #1 | P value |
|  | *N=9505* | *N=9505* |  |
| Age (years) – mean (SD) | 30.1 (4.7) | 26.4 (4.7) | <0.0001 |
| Age categories – n (%) |  |  | <0.0001 |
| <25 year old | 1094 (11.5%) | 3491 (36.7%) | <0.0001 |
| 25-30 year old | 3506 (36.9%) | 3665 (38.6%) | <0.0001 |
| 30-35 year old | 3231 (34.0%) | 1797 (18.9%) | <0.0001 |
| >35 year old | 1674 (17.6%) | 552 (5.8%) | <0.0001 |
| Health coverage assistance – n (%) | 2858 (31.0%) | 2627 (28.7%) | 0.0008 |
| Deprivation index quintiles – n (%) |  |  | 0.88 |
| 1 | 1001 (11.1%) | 994 (11.2%) | 0.92 |
| 2 | 1698 (18.9%) | 1637 (18.5%) | 0.45 |
| 3 | 1918 (21.4%) | 1876 (21.1%) | 0.75 |
| 4 | 2013 (22.4%) | 1990 (22.4%) | 0.99 |
| 5 | 2352 (26.2%) | 2374 (26.8%) | 0.39 |
| Diabetes (treated) at pregnancy – n (%)  Type 1 – n (%)  Type 2 – n (%) | 26 (0.3%)  119 (1.3%) | 12 (0.1%)  76 (0.8%) | 0.03  0.002 |
| Hypertension (treated) at pregnancy) – n (%) | 260 (2.7%) | 105 (1.1%) | <0.0001 |
| Sleep apnea syndrome (treated) – n (%) | 104 (1.1%) | 21 (0.2%) | <0.0001 |
| Nulliparous – n (%) | 10703 (100%) | 3285 (34.6%) | - |
| Medically assisted procreation – n (%) | 538 (5.7%) | 654 (6.9%) | 0.0006 |
|  |  |  |  |

Continuous variables were compared using paired t test. Categorical variables were compared using Mc Nemar Chi^2^ test;

BS: Bariatric surgery;

# Supplementary figures

## Supplementary Figure 1: Standardized difference of matching variables before and after matching


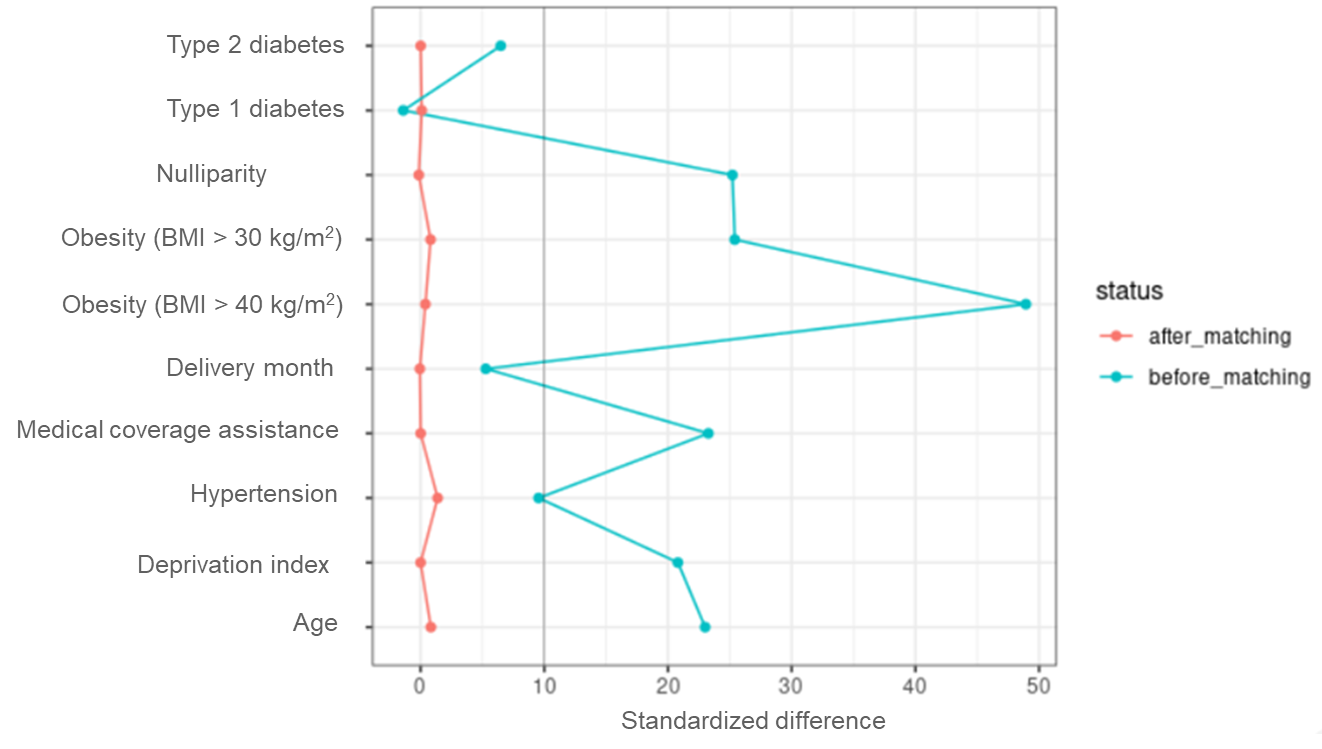


Standardized difference of matching variables between post-BS and control pregnancies before matching (blue) and after matching (red).

## Supplementary Figure 2: Adverse pregnancy and neonatal outcomes in pre-BS pregnancies #2 vs. #1 (conterfactual analysis)


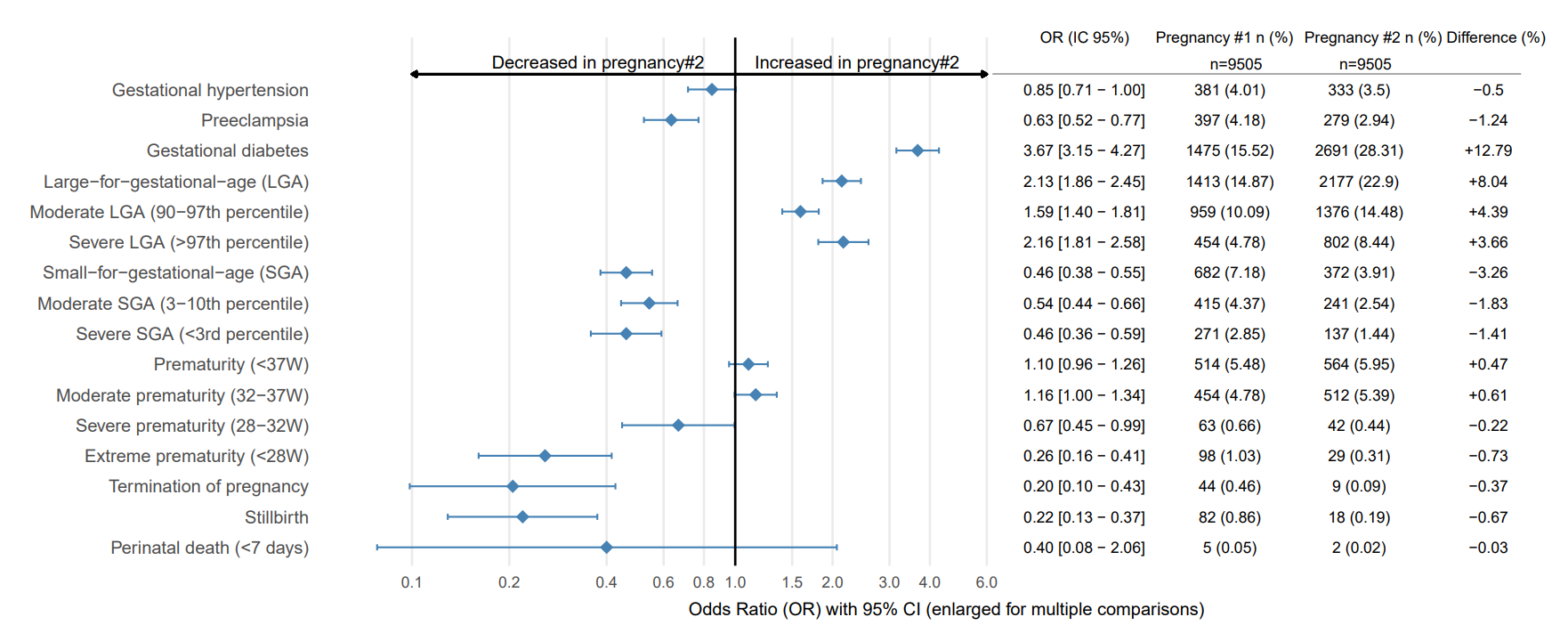


Occurrence of adverse pregnancy and neonatal outcomes of pre-BS #2 vs. pre-BS #1 pregnancies. Odds ratios are calculated from conditional logistic regression with the mother ID as a stratum. Confidence intervals are enlarged proportionally to the p-value increase after multiple comparison adjustment by using the false discovery rate method. BS: metabolic bariatric surgery; W: weeks of gestation

# References

1. Pina Vegas L, Drouin J, Weill A, Dray-Spira R. Pregnancy outcomes in women with rheumatoid arthritis: an 11-year French nationwide study. RMD Open 2024;10(1):e003762.

2. Ben Messaoud K, Bouyer J, de La Rochebrochard E. Infertility Treatment in France, 2008–2017: A Challenge of Growing Treatment Needs at Older Ages. Am J Public Health 2020;110(9):1418–20.

3. Billionnet C, Mitanchez D, Weill A, et al. Gestational diabetes and adverse perinatal outcomes from 716,152 births in France in 2012. Diabetologia 2017;60(4):636–44.

4. Johansson K, Cnattingius S, Näslund I, et al. Outcomes of Pregnancy after Bariatric Surgery. N Engl J Med 2015;372(9):814–24.

5. Simon E, Bechraoui-Quantin S, Tapia S, et al. Time to onset of cardiovascular and cerebrovascular outcomes after hypertensive disorders of pregnancy: a nationwide, population-based retrospective cohort study. American Journal of Obstetrics and Gynecology 2023;229(3):296.e1-296.e22.

1. [Croissance néonatale (audipog.net)](https://www.audipog.net/Applications-mobiles-croissance) [↑](#footnote-ref-1)
