# Supplementary material for: Maternal and neonatal outcomes of pregnancies after metabolic bariatric surgery: a retrospective population-based study
Source: Lancet Reg Health Eur. 2025 Mar 22;51:101263. doi: 10.1016/j.lanepe.2025.101263 (PMC11982497; doi:10.1016/j.lanepe.2025.101263)
Supplement: French abstract [file mmc2.docx]

**Résumé**

**Contexte :** L'incidence des grossesses après une chirurgie bariatrique (CB) métabolique post-opératoire est en augmentation. Les études précédentes ont fourni des résultats contradictoires concernant le risque de prématurité, de mortinatalité, de décès périnatal et le délai optimal entre la CB et la conception. Cette étude a examiné les issues maternelles et néonatales des grossesses post-CB ainsi que leurs facteurs associés.

**Méthodes :** Étude rétrospective nationale de toutes les grossesses post-CB en France entre le 1er janvier 2013 et le 31 décembre 2022. Comparaisons de 55 941 grossesses post-CB avec 223 712 contrôles appariés sur la date d’accouchement, la parité, l’âge, l’obésité, l’hypertension, le diabète et le statut socio-économique (ratio 1:4), en utilisant des équations d'estimation généralisées. Comparaison de 11 777 grossesses post-CB avec des grossesses pré-CB en par<< régression logistique conditionnelle.

**Résultats :** Les grossesses post-CB étaient associées à un risque réduit d’hypertension gestationnelle (odds ratio [OR] 0,57 [IC 95 % 0,53-0,62]), de prééclampsie (OR 0,59 [0,55-0,64]) et de diabète gestationnel (OR 0,64 [0,62-0,66]) par rapport aux contrôles. Des réductions de risque similaires mais plus marquées ont été observées par rapport aux grossesses pré-CB. Le risque de petit poids pour l’âge gestationnel (PAG) était augmenté (OR 1,74 [1,68-1,79]) par rapport aux contrôles et aux grossesses pré-CB (OR 1,88 [1,64-2,16]). Le risque de prématurité était accru (OR 1,27 [1,22-1,31]) par rapport aux contrôles mais non par rapport aux grossesses pré-CB (OR 0,95 [0,85-1,06]). Comparé aux contrôles, le risque de mortinatalité était augmenté (OR 1,2 [1,06-1,35]), médié par le PAG, tout comme le risque de décès périnatal (OR 1,5 [1,13-1,99]), médié à la fois par la prématurité et le PAG.

L’augmentation du risque de PAG par rapport aux contrôles était plus marquée en cas de malnutrition (OR : 2,38 [1,96-2,88], p_interaction_ <0,0001), lorsque l’intervalle entre la CB et la conception était inférieur à 6 mois (OR : 1,95 [1,72-2,21], p_interaction_ <0,0001) ou entre 6 et 12 mois (OR : 1,86 [1,70-2,04], p_interaction_ <0,0001), ainsi qu’avec un bypass gastrique (OR : 1,88 [1,77-2,00], p_interaction_ <0,0001). L’augmentation du risque de prématurité par rapport aux contrôles était plus marquée en cas de malnutrition (OR : 2,45 [1,99-3,00], p_interaction_ <0,0001) et de bypass gastrique (OR : 1,46 [1,36-1,57], p_interaction_ <0,0001).

**Interprétation :** La CB était associée à une réduction du risque de complications maternelles, mais à une augmentation du risque d’événements néonatals indésirables. Les risques de PAG et de prématurité sont plus élevés lorsque l’intervalle entre la CB et la conception est court, en cas de bypass gastrique et en présence de malnutrition. Les grossesses post-CB pourraient être considérées comme à haut risque, nécessitant un suivi nutritionnel et obstétrical rapproché.

**Financement :**
Soutien de l’INSERM et du Ministère français de la Santé (Messidore 2022 n°97).
